# Validity of Random Triglyceride Levels in Infants Receiving Parenteral Nutrition

**DOI:** 10.3389/fped.2021.601915

**Published:** 2021-06-17

**Authors:** Mohammad Y. Bader, Melanie A. Lam, Fernando Munoz, Leslie Thompson, Ranjit I. Kylat

**Affiliations:** ^1^Department of Pediatrics, University of Arizona Health Sciences, Tucson, AZ, United States; ^2^Banner University Medical Center, Tucson, AZ, United States; ^3^College of Pharmacy, University of Arizona, Tucson, AZ, United States

**Keywords:** neonate, parenteral nutrition, intravenous fat emulsions, triglyceride levels, hypertriglyceridemia

## Abstract

**Background:** Intravenous lipid emulsions (IL) are an important part of parenteral nutrition (PN) to meet essential fatty acid (EFA) requirements and metabolic demands of neonates and preterm infants. Some critically-ill neonates may not metabolize IL effectively which can lead to hypertriglyceridemia. Risks associated with this include increased pulmonary vascular resistance, displaced bilirubins, and platelet or macrophage dysfunction. Serum triglyceride (TG) concentration is used as a marker for lipid tolerance and predictor of potential complications involved with IL administration, but the clinical significance of this is still debated. Management of TG levels with regard to timing of laboratory tests, the ideal goal range, and duration of infusion of IL varies across institutions and is not standardized.

**Methods:** Single-center, retrospective study of newborn infants receiving parenteral nutrition (PN). Fasting and non-fasting TG levels were drawn during the same lipid infusion of 2–3g/kg/day. The primary outcome was the difference between fasting and non-fasting TG levels. Statistical assessment of continuous data was done with student *t*-test and nominal data was evaluated using X2-test and logistic regression.

**Results:** Forty infants were included with mean gestational age at birth of 29.5 ± 3.4 weeks and mean birth weight of 1.3 ± 0.5 kg. Mean time between lab draws while on same IL dose was 11.6 ± 0.2 h with resulting mean fasting and non-fasting (random) TG levels 82 ± 40 mg/dL (95% CI 68.4, 97.6) and 101 ± 40 mg/dL (95% CI 88.5, 115.8), respectively. Mean difference between TG levels during lipid-free interval and during infusion was −18.6 ± 51.2 mg/dL (95% CI −35.0, −2.3; *p* = 0.03).

**Conclusion:** We concluded there is no difference in the management of IL, when TG level was drawn randomly or as fasting sample. Obtaining TG level during routine lab draws is appropriate. We extrapolated that the administration of IL over 24 h will not interfere with TG level.

## Introduction

Parenteral nutrition (PN) is essential in critically ill neonates to prevent postnatal growth failure (1, 2). Intravenous lipid emulsions (IL) provide energy and essential fatty acids (EFA) to help these infants meet metabolic demands and achieve adequate postnatal growth (1–3). An increase of the cumulative intake of lipids during the first 2 weeks after birth has been associated with improved neurodevelopment at 1 year corrected age (4). Monitoring TG levels is essential as some extremely premature and critically ill infants may be at risk of developing hypertriglyceridemia. Complications associated with elevated TG levels include increased pulmonary vascular resistance, decreased pulmonary function, development of chronic lung disease, displacement of bilirubin, and platelet and macrophage dysfunction (5, 6). However, in majority of cases, early administration of IL is well-tolerated and has numerous benefits including improved post-natal growth, improved glycemic control, decreased rates of retinopathy of prematurity, and better long term neurodevelopmental outcomes (3, 6, 7). Serum triglyceride (TG) level is used as a marker for lipid tolerance and predictor of potential complications involved with IL administration. While several studies have evaluated the optimal infusion duration of IL in neonates, the optimal timing of TG measurement to evaluate for lipid tolerance has not been clearly determined. The aim of this study was therefore to evaluate for significant differences in serum TG levels when measured during fasting (lipid-free interval) and non-fasting (during IL infusion) intervals in infants receiving IL.

## Methods

This is a retrospective study of infants admitted to the neonatal intensive care unit (NICU) at Banner University Medical Center and University of Arizona, Tucson between December 2018 and June 2019. Infants who were admitted to the NICU, started on parenteral nutrition (PN), and had both random and non-random TG levels drawn on the same day and on the same IL dose were included in the study. Our NICU practice was to initiate IL as Intralipid 20% at 1g/kg/day and increase the dose by 0.5–1 g/kg/day to achieve a goal of 3g/kg/day. To facilitate tolerance, our NICU infused IL over 20 h with a lipid-free (“fasting”) interval of 4 h. At the end of this “fasting” interval, TG levels were drawn at this precise time to prevent confounding factors that might lead to falsely elevated TG levels. Patients who were previously on full enteral feeds, have metabolic disorders, or have sepsis were excluded. The institutional ethics and review board approved the study.

The primary outcome was the difference between fasting and non-fasting TG levels drawn during the same lipid infusions of 2 or 3 g/kg/day. To compare these differences, random “non-fasting” TG levels were obtained along with routine laboratory tests which were drawn, between 4:00–5:00 AM, while the patient was receiving the IL infusion. The dose of IL was titrated to a goal of 3g/kg/day if the prior fasting TG levels were within the normal range. For any TG level >250 mg/dL, the IL was decreased by 1g/kg/day.

We sought to determine if fasting and non-fasting TG levels varied significantly based on gestational age, birth weight, time to start of enteral feeds, time to reach full enteral feeds, total duration of PN, direct bilirubin level, and timing between assays. Continuous data were analyzed using sample *t*-test with a confidence level of 95% (α = 0.05), and power (β) of 0.8, p of < 0.05 determined statistical significance. X (2) test and Mann -Whitney U test was used for non-parametric data analysis. One-way and two-way analyses of variance (ANOVA), and logistic regression were performed.

## Results

### Demographics

**Figure 1:**
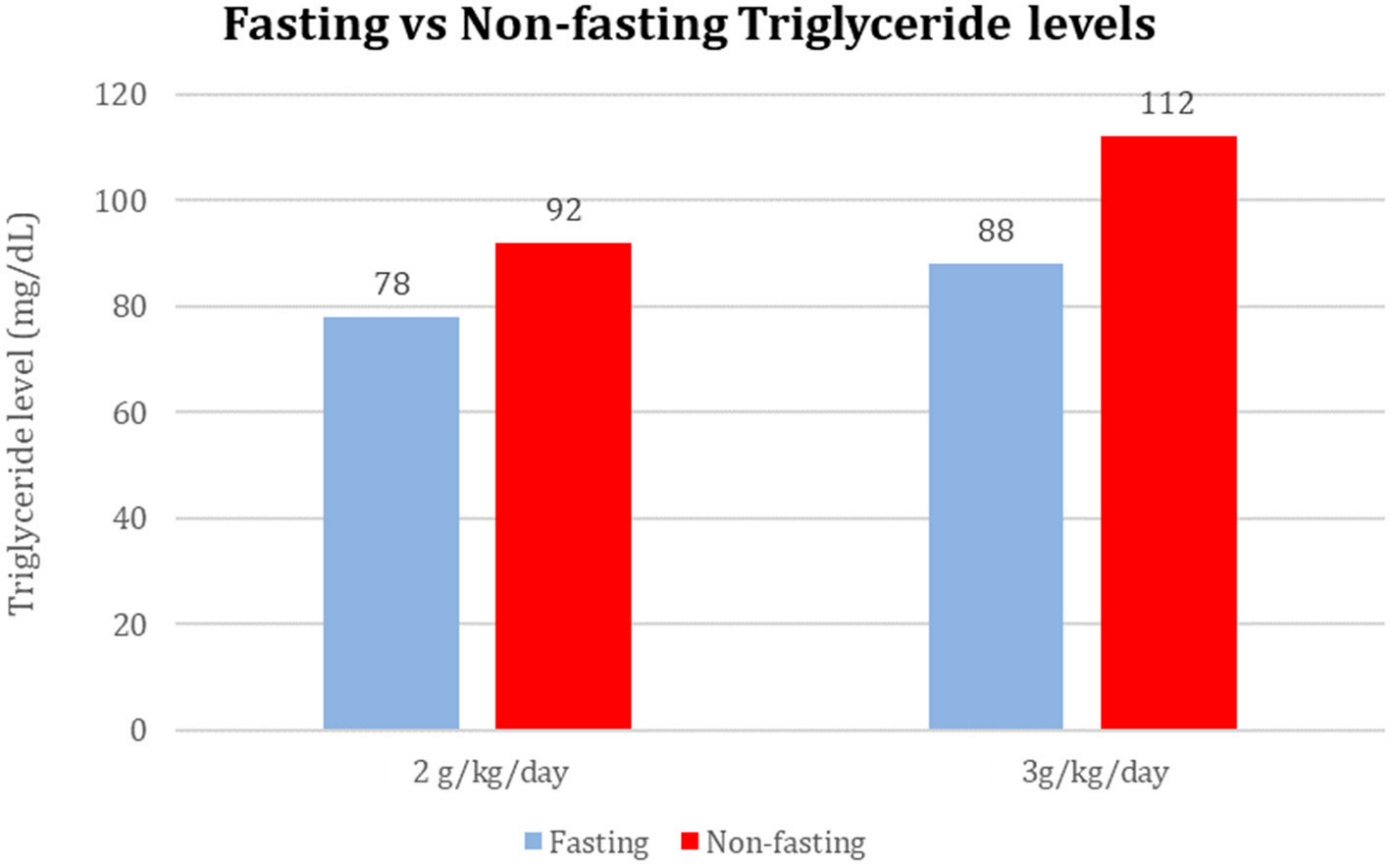
Mean fasting and non-fasting serum TG levels in infants receiving 2 and 3 g/kg/day.

**Figure 2:**
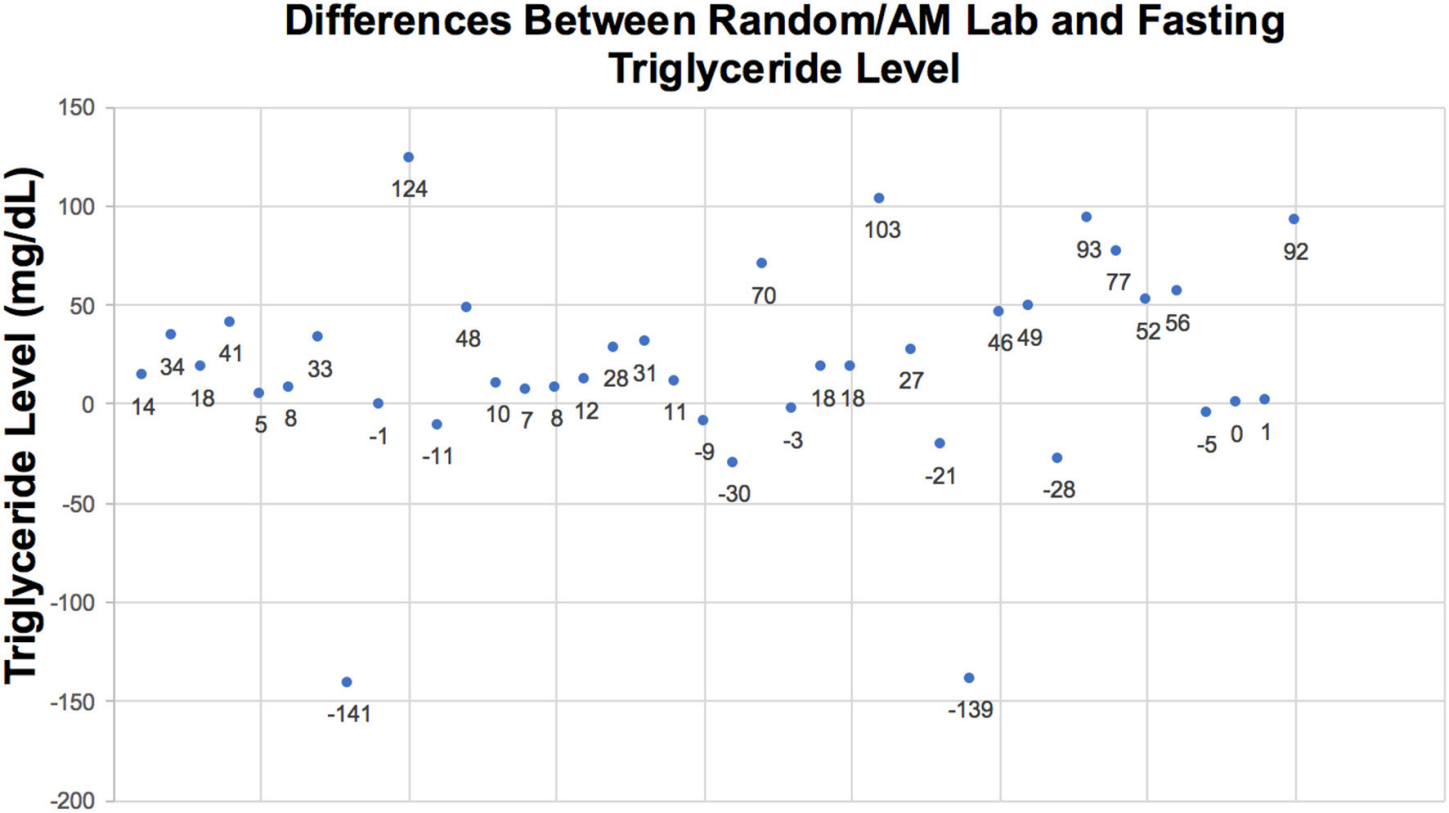
Difference between all non-fasting and fasting TG levels at 2 and 3g/kg/day.

The study included 40 patients admitted to the NICU and received 20% IL infusion at either 2 or 3g/kg/day. Patient demographics are shown in Table 1. 22 patients were male and 18 were female. Mean gestational age at birth was 29.5 ± 3.4 weeks (*p* = 0.745), with 13 patients (33%) born at <28 weeks, 16 patients (40%) born at 28 to 32 weeks, and 11 patients (28%) born at 33 to 37 weeks. Of the 40 samples, 13 were in patients weighing < 1 kg, 13 in 1–1.5 kg, and 14 in >1.5 kg (Figures 1, 2). Mean birthweight was 1.3 ± 0.5 kilograms. Mean direct bilirubin was 0.86 (range 0.2, 7.4) and the highest direct bilirubin was 1.132 mg/dL (range 0.2, 7.4). Mean time to reach full enteral feeds was 15.6 ± 0.8 days.

**Table 1:** Demographics of all infants.

**Characteristic**	***N* = 40**	
Sex, *N* (%)	Male	22 (55)
Birth Weight, *N* (%)	<1,000g	11(28)
	1,000–1,500g	13 (32)
	>1,500g	16 (40)
Birth weight Mean (Kg)		1.3 ± 0.6 (0.65, 3.0)
Mean ± SD (Min, Max)		
Gestational Age (weeks)		29.5 ± 3.4 (24.2, 35.3)
Mean ± SD (Min, Max)		
Direct Bilirubin (mg/dL)		0.86 (0.2, 7.4)
Mean (Min, Max)		
Highest direct bilirubin (mg /dL)	Mean level (mg/dL) (Min, Max)	1.13 (0.2, 7.4)
Mean (Min, Max)		
Nutrition status	Mean duration of parenteral nutrition (days from admission)	13.2 ± 5
	Mean ± SD	
	Mean time to start enteral feeds (days from admission)	2.2 ± 1
	Mean time to reach full enteral feeds (days from admission) ± SD (Min, Max)	15.6 ± 0.8 (10, 29)

### Triglyceride Levels

Among 40 sample pairs of TG levels, 22 were drawn when infants were receiving IL at 2 g/kg/day and 18 reflected levels drawn when IL was infused at 3g/kg/day. TG levels are shown in Table 2. Mean time between lab draws while on same IL dose was 11.6 ± 0.2 h. Fasting (after 4 h IL free period) and non-fasting (random) TG levels for the 2g/kg/day group ranged from 28 mg/dL to 190 mg/dL and from 49 mg/dL to 276 mg/dL, respectively. Fasting and non-fasting TG levels for the 3g/kg/day group ranged from 39 mg/dL to 260 mg/dL and from 57 mg/dL to 180 mg/dL, respectively. A TG level above 250 mg/dL was measured once in the non-fasting 2g/kg/day group and once in the fasting 3g/kg/day group. Mean fasting and non-fasting TG levels for all patients were 82 ± 40 mg/dL (95% CI 68.4, 97.6) and 101 ± 40 mg/dL (95% CI 88.5, 115.8). Mean fasting and non-fasting TG levels for patients receiving IL at 2g/kg/day were 77.8 ± 9.7 mg/dL (95% CI 58.3, 97.4) and 91.9 ± 9.0 mg/dL (95% CI 73.6, 110.2). Mean fasting and non-fasting TG levels for patients receiving IL at 3g/kg/day were 88.2 ± 10.7 mg/dL and 112.4 ± 9.9 mg/dL (95% CI 92.2, 132.7). The mean difference between TG levels during lipid-free interval and during infusion were −18.6 ± 51.2 mg/gL (95% CI −35.0, −2.3; *p* = 0.03). Subset analysis in the 2 and 3 g/kg/day groups showed no statistical difference with p of 0.32 and 0.09, respectively.

**Table 2:** Fasting and non-fasting TG levels at 2 g/kg/day, 3 g/kg/day IL infusions and when combined at either infusion rate.

	**IL free (fasting) TG (mg/dL)**	**Non-fasting TG (mg/dL)**	***p*-value**
2g/kg/day	78 ± 40	92 ± 47	0.32
3g/kg/day	88 ± 51	112 ± 36	0.09
Total (2 or 3 g/kg/day combined values)	83 ± 40	101 ± 40	0.03 (CI 95% 25.5–50.6)

## Discussion

IL administration is a critical part of optimal PN in critically ill infants' especially very low birthweight infants (VLBW) who are susceptible to EFA deficiency. Dose of lipids administered is based on EFA goals and daily caloric needs. Providing adequate EFA is vital to ensuring cell membrane structure, cellular pathway signaling, and promoting appropriate gene expression. Despite the common practice of providing IL in neonates, there still lacks consensus for evaluating lipid tolerance with regards to appropriate timing of TG levels. Variation in practice include – some institutions' preference for specifically timed TG levels after completion of 20 or 24 h IL infusion. Other institutions may prefer drawing TG levels with routine labs while IL is infusing.

Multiple studies have demonstrated that continuous lipid infusion over a 24 h cycle is safe in neonates and does not result in hypertriglyceridemia when lipids are infused at 2–3 g/kg/day (8, 9). Furthermore, use of a shorter infusion time and lipid free interval has not been shown to improve TG clearance or to reduce the risk of hyperbilirubinemia (10, 11). A study by Kao et al. has shown that premature neonates experienced less fluctuations in serum lipid levels and less hypertriglyceridemia when receiving a continuous vs. intermittent infusion (5). Although there is a trend in clinical practice toward continuous IL infusions, the optimal timing of TG measurement is still not clearly established. Morris et al. found that TG levels decrease most rapidly in the 4 h after stopping IL infusion, suggesting that the best time to check for lipid tolerance would be after this lipid-free interval (12).

The goal of our study was therefore to evaluate for a significant difference in serum TG levels when measured during a lipid-free interval (fasting) and during infusion, to determine if levels checked during a continuous infusion provide an accurate and acceptable estimation of lipid tolerance. In our study, we found that the mean fasting TG level was significantly lower than the non-fasting level at 2 and 3 g/kg/day combined (*p* 0.03). As the threshold TG level of 250 mg/dL was reached only once in each of the fasting and non-fasting groups, the difference in fasting and non-fasting TG level did not ultimately change clinical management for most patients. Therefore, our institution's practice of drawing specifically timed TG levels during 4 h of lipid-free interval (after 20 h infusion), a lipid free, fasting interval prior to measurement of TG levels may not be necessary, and random, non-fasting serum TG measurements may provide an acceptable depiction of lipid tolerance. This may lead to simpler timing of lipid administration and TG measurement. The use of random, non-fasting TG measurements decreases the frequency of blood draws, decreases amount of blood volume collected, and decreases rate of accessing central lines and ultimately reducing potential risk of infection.

The monitoring of IL is particularly important in premature and VLBW infants as they have been shown to metabolize lipids more slowly due to their reduced lipoprotein lipase (LPL) activity. These patients will have relatively higher TG levels due to decreased lipid tolerance (1, 3, 5, 13, 14). Earlier studies suggest that premature infants better tolerate IL (defined by serum TG levels < 250 mg/dL) when administered over 24 h (15, 16). The ability to assess lipid tolerance in these high-risk populations with random, non-fasting TG measurement provides an optimal alternative in these fragile patients who are at relatively higher risk of morbidity associated with anemia, blood transfusions, and infection related to central line access.

An important limitation of this study is the relatively small sample size of 40 patients. It is possible that a larger study would have detected more abnormal TG levels, specifically in the random TG measurement group. The majority of patients in the study (72%) were < 32 week GA, 28% were 32 and above so this could have skewed the results toward improved lipid tolerance. Infants born after 32 weeks of gestation were shown to have improved lipid clearance (17). Implications associated with decreased ability to clear intravenous lipid is due to immature heparin-induced lipoprotein lipase activity and hepatic immaturity putting more premature and VLBW neonates at risk of higher plasma TG levels. Therefore, future studies enrolling a larger and more homogenous sample of VLBW infants is warranted to determine optimal timing and frequency of obtaining TG levels.

## Conclusions

Random, non-fasting serum TG levels with routine labs may provide an adequate assessment of lipid tolerance and help guide management in neonates receiving IL. Potential benefits to include simpler IL infusion times, TG measurements, decreased frequency of blood draws, and decreased central line access.

## Data Availability Statement

The original contributions presented in the study are included in the article/supplementary material, further inquiries can be directed to the corresponding authors.

## Ethics Statement

The studies involving human participants were reviewed and approved by IRB, University of Arizona and was provided a waiver and deemed to be no more than minimal risk.

## Author Contributions

All authors listed have made a substantial, direct and intellectual contribution to the work, and approved it for publication.

## Conflict of Interest

The authors declare that the research was conducted in the absence of any commercial or financial relationships that could be construed as a potential conflict of interest.
